# Suggestions and Kind Words from Friends

**Published:** 1872-10

**Authors:** 


### SUGGESTIONS AND KIND WORDS FROM FRIENDS.

"I, for one, thank your publisher for his kind indulgence, and also for sending me my bill. I assure you I pay for your journal most cheerfully. I would not be deprived of it for four times the price of subscription, as it fills the bill more nearly than any journal of my acquaintance. I am pleased—delighted—with your plans for the future. I am now certain of its success. My September number came securely wrapped, and my address was written plainly—which I esteem a favor. Send all like this, and I shall not be one of the complainers. You know much of your success depends upon putting up your journal securely, and having it properly directed."

We believe very often our journal has failed to reach subscribers from the causes set forth by our correspondent.

The September number referred to was put up and forwarded by our business manager, who will, in future, see personally to the mailing department. We feel confident mistakes in future will be few, from the well known business qualifications of our manager. Friends can now go to work, feeling secure of having the journal sent promptly. Our business manager asks for the co-operation of all subscribers, and desires that each and every one of them shall act as Agent for the COMPANION, in securing subscribers, etc. Go to work, friends, and swell the subscription list to the utmost.

"I READ with great interest and pleasure the remarks of your correspondent in your last number, in relation to the duties of the profession in taking a bold stand in repudiating the pretentious claims of inferior Medical Colleges to patronage. I indorse all he has written—and written so truthfully and well. The good men will hail with joy the day when these so-called colleges shall be expunged from the list of Medical Institutions. Ask your correspondent, who so ably advocates correct principles, to continue the fight. I think you would do well to re-publish the article in your next. It is pointed and truthful, and will do great good."

"I see in your last number you think of changing the name of your excellent journal. This will be a move much to its benefit. So long as the affix "Georgia" remained, it seemed to localize the COMPANION as a State enterprise; when it should be by title as design, more comprehensive in name. It has, I am glad to know, already become as much the journal of other States as of Georgia, and the profesion will be pleased to see the evidence you show of making it the journal of the entire medical fraternity irrespective of State lines. I trust, therefore, that your Associates and readers will at once respond to your generous request to name their "organ." By all means urge them to send in the names at once that the committee may select the future name of the journal by the first of the new year. T."

OUR journal is the organ of no "ring or clique"—but of the profession. Every gentleman who feels interested in it will please suggest a name, as our only object is to serve the profession in name and matter. As stated before, a committee of our Associates will, from the various names suggested, select one which they may think best. Our friend "T" will do us and the profession a favor by writing for our journal often. We hope he will.

---

"MAY I not most kindly suggest that no further notice be given in your valuable journal to the "Gaillard correspondence"? This you can well afford to do, having fully vindicated the truth and yourself to the mind of the public.

"As a champion of truth in our profession, you need not be driven to measure swords with every individual who may delight in notoriety even when gained unrighteously—but let a big-hearted charity be *silent* and forgive, for truth is mighty and has prevailed. M. D."

THE Senior Editor appreciates the above, coming, as it does, from a gentleman whose heart always beats responsive to truth and justice. It is to be hoped that *now* an end has been reached, of a controversy which has been thrust upon this journal and its senior editor, contrary to its and his desires. Therefore, we assure our correspondent, we shall not renew the controversy unless compelled to do so in vindicating character and the truth of history. Every gentleman who has character is conscious of its value and hence the duty of defending it when assailed. Those who never possessed it do not feel this necessity; and regard such discussions of small importance. Like our correspondent, we think enough has been said for the vindication of character and the Senior Editor can *now* rest on his oars, and act only as may be determined for the good of society and the honor of the profession.

---

"I always felt, from the first number of your journal, I had the pleasure of seeing, that it could not fail to succeed—from the plan upon which it was arranged, and the condensation of medical information it placed so happily and conveniently in the hands of the practitioner. However, lately I have had some misgivings as to its permanency, from the fact of the irregularity of its publication, as to time. True, every number has come to me, but many of them were delayed so long as to make me rather sad with the reflection that the best journal of my knowledge would, like so many others started South, be compelled, finally, to suspend. But these impressions have been removed by your late number, in which I find your letter to subscribers. I am convinced more than ever of the justice, as well as the necessity, of your placing it strictly upon a Cash system—as the only plan upon which any publication can now be made permanently successful. I am delighted at this determination on your part, and all true friends to the enterprise will say—amen!

"As you know, I have had some experience in editing and publishing, I can fully appreciate your trials and difficulties, more especially now when prices of material and labor have reached the "high-water mark." For all these the publisher must pay cash—promptly, or down goes the whole fabric. In no department of business is "cash" so imperatively demanded, so absolutely necessary, in order to meet the daily expenditures. Truly the path of the publisher is not strewn with roses—a fact delinquent subscribers do not appreciate or realize. If they could they would so fully appreciate the necessities of the publisher that they never would permit him to dun them—never subject him to annoying delays and the additional expense of appealing to them by letter and otherwise.

"Knowing all this, as I do, permit me to make a few suggestions: 1st. Let every subscriber understand your difficulties and the necessity forced upon you by these, to adopt the Cash basis. 2d. Under the operation of the Cash plan, you should always keep on hand, or have arrangements by which you can promptly secure the *very best* materials—as paper, etc. 3d. To bind the publisher, by contract, to execute his part in the best style, and to have each number issued promptly on the day agreed upon. 4th. Your business manager should see that the journal should be put up substantially, the *names*, *post-offices*, *counties* and *State* written plainly on envelopes. 5th. You should, therefore, request all subscribers to give their names, post-office, counties and State plainly. 6th. That you request every subscriber to inform his post-master that he is a subscriber to your journal—giving its name, and that it ought to reach his office at a certain time.

"If you will carry out these suggestions I *know* you can furnish our profession one of the most "wide-awake" journals in the country. One that will reflect credit upon you and them and be the means of doing much for the weal of our cause and of the science upon which it is based. Hopeful of your future—knowing your energy and zeal—you have only to push on in the right way, animated by professional honor in the future as in the past, to make your journal among the foremost of the Union. Believing you will do all this, and doing it reap a large harvest of success, I am, etc., * * *."

We publish the above with the greatest pleasure. It comes up to our ideas so completely—so fully, that we shall adopt the suggestions of our friend heartily and zealously. We are conscious no other plan save the Cash system can give that full measure of success to our enterprise we and our readers desire. Hence, that plan ought to, and will, we think, meet their concurrence, endorsement and support. The suggestions in regard to the business management, are happy and correct, and we will promise our friend and readers to see them faithfully executed. Our friend is sanguine in behalf of our journal. Truly he has reason to be so, for we assure him the circulation of the COMPANION falls short of but few Medical Journals in the Country. If its friends will second the suggestions of our kind advisers, it will, before the lapse of another year, attain the head of the list. We are not insensible of the flattering encomiums of our friend. It shall be our aim to fully merit all he has said from the profession generally. If the end shall justify and confirm his opinions of its merit, our gratification shall be measured alone by the good it may accomplish and the truths it may enforce.

---

"Editors and readers, I think, rarely turn over in their minds, with sufficient study, the one question I wish to propound, to-wit: What are the true objects of a Medical Journal?

It is very easy to say what *are not* the objects in legitimate medical journalism. We all know it is the duty of the brotherhood, battling in all countries and climes, to clasp hands before the altar of Truth in the Temple of Science. To spurn error; to reject falsehood; to dig patiently for the precious ore of truth; to investigate, explore; to extort nature's secrets by an earnest, diligent, constant appeal to facts as God has arranged them, and as man must find them. Thus engaged in a cause holier than all other earthly employments, the physician, true to himself and science, has two grand objects in view: First. To open up the labyrinths of nature to science. Second. To make these facts available in the interests of mankind. These then, being the objects of the physician, they become the *only* true objects of those making his literature. It is no part of a medical journal to elaborate theories upon theology or politics, or to discuss questions arising therefrom, save only when the disciples of these invade the realm of medical science to subvert its truths and to inflict injury upon humanity thereby.

"But what are the true objects of a Medical Journal? These "objects" may, for convenience of argument, be placed in four great divisions, under which, to every reflecting mind, will fall many sub-divisions:

1st. Things that relate to its scientific literature proper.

2d. Things that relate to the government and morals of the brotherhood.

3d. The relationship depending between the arts and medicine.

4th. The mutual dependence of the disciples of these to each other.

"With your permission, I will try, briefly, to show the value of these. Under the first division it is the duty of a medical journal to lay before its readers the ever increasing truths being developed by the scientific explorers of the great brotherhood; to separate the false from the true by the strictest analysis of facts; to unfold, briefly as possible, the practical truths of medicine, founded upon the tested experience of the common brotherhood; to furnish the most scientific deductions for correct practice based upon the latest physiological and pathological developments, with the best therapeutical means the entire field of nature may afford, guided by these developments and experience; to cull the practical thoughts embodied in communications, from clinics, hospitals, and formulæ of the masters of our art—as exhibited in the medical literature of both hemispheres, to serve as a medium through which the toilers of our art may communicate and discuss all scientific topics, with the brotherhood, connected with the weal of the profession.

"Under the second division, the object of a medical journal should be to "stir up" the lukewarm and seek to concentrate the affections of the brotherhood upon every measure for the advancement, honor, dignity and value of true medicine; to give tone to the profession by appeals to professional honor and the enforcement of medical etiquette; to exhort the brotherhood to good works and strict allegiance to professional morality as taught in the "Book of Books" and epitomized in the Code of Ethics; to inculcate, day and night, by pen and voice, the important duty, as a rock of safety and defence, of the fraternity clasping hands in solemn compact to dignify medicine by exalting the individual status of the brotherhood, by education, pure morals and brotherly love; to advocate individual integrity in a common cause, that by our united effort uneducated and unworthy men may not find a preceptor in all the brotherhood, or an entrance through the gates of our medical schools; to frown down with professional displeasure schools of cross-road villages, destitute alike of scholarship or the facilities of teaching.

"I would remark here, that the standard of Medical Education can never be raised so effectually as by the profession, *en masse*, adopting the suggestions which from time to time have appeared in "our" COMPANION, viz: That practitioners everywhere should refuse, unrelentingly, to receive immoral or uneducated students in their offices for instruction; that they should, when preparing students, require them, as a part of the contract between Preceptors and Students, to pledge themselves to attend, from the start, only schools of standing and reputation; that colleges recognized as true representatives of medical learning by the profession should refuse any, the slightest, recognition of those schools which have and still persist in lowering the educational standard.

"Under the third head, it is the duty of a Medical Journal to open an advertising department, through which every business, legitimately connected with the profession may be presented to its readers for the purpose of assisting the practitioner by all the appliances of his art in his efforts to combat disease. Through such a channel alone can the practitioner arrive at any degree of certainty in supplying his wants. If none but honorable men be admitted in the advertising department, the practitioner can feel secure. This, I am glad to know, has been the course of the COMPANION. The practitioner must have—he feels the necessity of having—pure drugs, chemicals and the best instruments, from approved houses. They too, must have standard works which have stood the test of rigid criticism. They require many other things which they ought to find brought before the profession in a medical journal. I, therefore, say the COMPANION has inaugurated a movement in these respects, which has been long needed—a fact which should secure for it the thanks of every practitioner in the land.

"Under the fourth head, the relationship existing between those who furnish materials and appliances and the physicians should be mutual and honorable, as all should have, independent of business, the good of suffering humanity in view. It is unnecessary to dwell upon this, as all will perceive its importance at a glance.

"I am delighted with the course of your journal. Put it fully up to these objects and it will accomplish more than its warmest friends have ever dreamed. That it may do all this, and doing succeed, I ardently wish and expect."

It is almost unnecessary to say, the above has our *full* endorsement. The objects set forth by our correspondent, which ought to control the management of every Medical Journal, are many. We think they are legitimate and correct. We may not come up, fully, to the standard required, yet we promise our friend to keep his suggestions in view, and to do our best. We thank him for the kind endorsement he has given the COMPANION. Its course in the future will be no less earnest and faithful in meeting the every-day wants of our readers who are largely of the class of "busy practitioners." If they, like our correspondent, will "hold up our hands" and do their *best* for the COMPANION, it will do *its best* for them. It is their medium, and they can make it all they desire. We believe they will do it.

---

A Georgia friend writes:

"Success to your excellent COMPANION. Pay no attention to your jealous cotemporaries. Their anathemas will most likely recoil on their own heads."

A friend from another State writes:

"It seems to me, from my stand-point, that a "masterly inactivity" would be the line of action indicated in regard to Dr Gaillard and "the largest Medical Monthly in America." The rebound of Gaillard's guns will effectually "do" him, without the necessity of the COMPANION'S going out of its way to even notice him."

Another friend writes:

"Never print Dr. Gaillard's name again in any controversy. We are all tired of it."

Another friend writes:

"Place the subscription up to $3 00, the very least that a medical journal can be published for." * * * "We of the profession have no need of examples of controversy except on medical subjects of a controversial nature, and not much of these either, but facts, real, unvarnished facts, that will place us a step forward in our profession."

We thank our friends. Personal controversies are objectionable at all times, and yet it frequently becomes necessary for almost every one to defend his principles or his character. When these are assailed, no man ought to dodge the issue, and tamely submit to any unjust imputations. There was an effort made, we think, to ruin the Companion. Every means, it appears to the writer, was employed to accomplish that end. The effort has failed. We appreciate fully the kind expressions of our friends. We are rejoiced to be able now to say our "controversy" has ended. We shall not renew it. In future the Companion will be devoted wholly to its legitimate objects so fully set forth by a correspondent in another place. We invoke our friends, everywhcre, to aid us. With their generous help, and kind encouragement, we hope to make the Companion—under a new name—all they expect and we so ardently desire.

